# Growth patterns for untreated individuals with MPS I: Report from the international MPS I registry

**DOI:** 10.1002/ajmg.a.61378

**Published:** 2019-10-22

**Authors:** David Viskochil, Lorne A. Clarke, Luisa Bay, Hillary Keenan, Joseph Muenzer, Nathalie Guffon

**Affiliations:** ^1^ Department of Pediatrics, Division of Medical Genetics University of Utah School of Medicine Salt Lake City Utah; ^2^ British Columbia Children's Hospital Research Institute University of British Columbia Vancouver British Columbia Canada; ^3^ Hospital Nacional de Pediatría J. P. Garrahan Ciudad Autónoma de Buenos Aires Argentina; ^4^ Sanofi Genzyme Cambridge Massachusetts; ^5^ Department of Pediatrics University of North Carolina at Chapel Hill Chapel Hill North Carolina; ^6^ Centre de Référence des Maladies Héréditaires du Métabolisme Hôpital Femme Mère Enfant Lyon France

**Keywords:** disease‐specific growth curves, lysosomal storage disorders, mucopolysaccharidosis, natural history

## Abstract

Mucopolysaccharidosis Type I (MPS I), caused by deficiency of α‐L‐iduronidase results in progressive, multisystemic disease with a broad phenotypic spectrum including patients with severe (Hurler syndrome) to attenuated (Hurler–Scheie and Scheie syndromes) disease. Disordered growth is common with either phenotype. The study objectives were to construct sex‐ and age‐specific estimated length/height and head circumference growth curves for untreated individuals with severe and attenuated disease and compare them with clinical reference standards. Untreated individuals in the MPS I Registry with at least one observation for length/height and/or head circumference and assigned phenotype as of May 2017 were included. Median growth for 463 untreated individuals with severe disease deviated from reference growth curves by ~6 months of age and fell below the third percentile by 4 years of age. Median head circumference was above reference curves from 3 to 4 months through 3 years of age. Among 207 individuals with untreated attenuated disease, median height fell below the third percentile by 9 years of age with divergence from reference curves by 2 years of age. MPS I‐specific growth curves will be useful in evaluation of long‐term outcomes of therapeutics interventions and will provide a foundation for understanding the pathogenesis of skeletal disease in MPS I.

## 1 INTRODUCTION

Documentation of the natural history of rare inherited metabolic disorders is an important component in the development and evaluation of therapeutics. Disordered growth is a common manifestation of the mucopolysaccharidosis (MPS). Disease‐specific growth charts are available for several MPS disorders (Montano, Tomatsu, Brusius, Smith, & Orii, 2008; Patel et al., 2014; Quartel et al., 2015) but have not been developed for MPS I. Describing the natural history of growth patterns in MPS I could prove helpful in the evaluation of treatment impact and provide insights into disease pathogenesis for this condition.

MPS I occurs in approximately 1/100,000 live births (Giugliani, Federhen, Michelin‐Tirelli, Riegel, & Burin, 2017; Moore, Connock, Wraith, & Lavery, 2008) and is caused by deficiency of α‐l‐iduronidase (IDUA) resulting in altered catabolism of the glycosaminoglycans (GAGs), dermatan sulfate and heparan sulfate (Muenzer, 2012). These catabolic defects lead to GAG accumulation as well as to the alteration of many secondary cellular pathways. Through pathophysiologic processes that are not well understood, patients present with progressive, multisystemic disease with variable age of onset and rate of disease progression. Disease phenotypes fit within a continuous spectrum of severity ranging from severe (Hurler syndrome, OMIM 607014) to attenuated (Hurler–Scheie OMIM 607015 and Scheie syndromes, OMIM 607016). Key features that define the spectrum include early manifestations and progressive neurocognitive impairment in the severe form of the disease, Hurler syndrome, to little or no cognitive involvement and later onset of disease‐specific manifestations in individuals with attenuated disease, Hurler–Scheie and Scheie syndromes.

Growth abnormalities are key manifestations of all of the mucopolysaccharidoses as well as many other skeletal dysplasias caused by genetic defects in glycosaminoglycan synthesis, such as hereditary multiple exostoses (Zak, Crawford, & Esko, 2002), Desbuquois dysplasia type 1 (Paganini, Monti, et al., 2019), among others (Mizumoto, Ikegawa, & Sugahara, 2013; Paganini, Costantini, et al., 2019). These clinical observations emphasize the importance of glycosaminoglycan homeostasis for normal growth (Clarke, 2011). The objective of this study was to construct sex‐ and age‐specific estimated length/height and head circumference growth curves for untreated individuals with either severe or attenuated MPS I to provide a basis for the evaluation of therapeutic interventions and pathogenic mechanisms.

## 2 METHODS

### 2.1 Editorial policies and ethical considerations

The study was approved by institutional review boards or ethics committees as required. Written informed consent from patients and/or their parents/guardians was obtained in compliance with all local laws and regulation.

### 2.2 Study design

The MPS I Registry (http://clinicaltrials.gov NCT00144794) is a voluntary, observational global longitudinal database established to capture standard of care clinical and biochemical assessments of MPS I and to evaluate clinical outcomes. Individuals are referred to the MPS I registry by their clinician and data are collected both retrospectively and prospectively as previously described (Pastores et al., 2007).

The registry is sponsored by BioMarin Pharmaceutical Inc. (Novato, CA) and Sanofi Genzyme (Cambridge, MA) and is overseen and directed by an independent Board of Advisors comprised of physicians who are experts in the care of MPS disorders.

### 2.3 Study population

Observations from untreated MPS I individuals in the Registry with at least one measure for length/height and/or head circumference and assigned phenotype as of May 2017 were eligible for inclusion in the analyses. For an observation to be included, data points must have reported units in an appropriate range (see below) and be linked with a date of diagnosis, date of height measure date of birth, and sex. Individuals with severe MPS I who had a natural history follow‐up time of greater than 12 years were excluded from analyses (*n* = 49).

### 2.4 Statistical analysis

Individuals were categorized with either “severe” (Hurler Syndrome”) or “attenuated” (including both Hurler–Scheie and Scheie Syndrome) MPS I. Data were stratified by reported sex and age (males 0–24 months; females 0–24 months; males 2–20 years; females 2–20 years) for the analysis of length/height observations. For head circumference, data were divided by reported sex and measures taken between 0 and 36 months of age, inclusive, consistent with the World Health Organization (WHO)/Centers for Disease Control and Prevention (CDC) reference data.

Observations four standard deviations beyond the median in 3‐month intervals (e.g., 0–3 months, 3–6 months) were excluded for those 0–24 months of age, and a 2 year interval was used for the 2–20 year range. This resulted in the exclusion of one observation from the 0–24 months range and two observations from the 2–20 years range. Head circumference measurements that were less than the preceding measurements were removed, as were measurements in excess of 58 cm (*n* = 1).

Descriptive analyses were performed on variables of interest to determine distribution and appropriate statistics. Continuous variables are expressed as medians and interquartile range (IQR: 25%, 75%), and as counts and frequency *n* (%). Age‐ and sex‐stratified standardized growth and head circumference data were downloaded from the National Center for Health Statistics (NCHS) for the WHO and CDC growth and head circumference charts (DepartmentHHS, 2002).

The General Additive Models for Location Scale and Shape (GAMLSS) of the R package was used to estimate the median, 5% and 95% curves for stature and head circumference for the respective age, sex, and phenotype strata from the natural history data of the MPS I Registry (Flegal & Cole, 2013). The distribution of measurements as a function of age is summarized by three parameters: skewness power in the transformation (L), the median (M), and coefficient of variation (S). The Lambda Mu Sigma (LMS) method was chosen because it allows equal weight per observation, regardless of the number of measurements per individual, and it provides results similar to those used by the NCHS (Flegal & Cole, 2013). Additionally, scatterplots were composed of the MPS I natural history data over the WHO and CDC reference charts using SAS v9.4 (Cary, NC). The head circumference chart was extended to 58 cm to accommodate the larger occipital frontal circumference (OFC) values seen among individuals with severe MPS I.

## 3 RESULTS

### 3.1 Participants

Among all participants in the MPS I Registry from October 2003 to May 2017, 670 had usable length/height observations prior to treatment intervention. MPS I was diagnosed by enzyme level in 443 patients, by genotype in 20, and by both enzyme level and genotype in 122 patients. Eighty‐five patients had no records available regarding method of confirmatory diagnosis; however, phenotype designation data were entered by health‐care providers familiar with and actively treating individuals with MPS I. There were 463 (69%) individuals categorized with severe and 207 (31%) with attenuated disease. Table 1 shows the median natural history follow‐up time and the median age at diagnosis for individuals with either severe or attenuated MPS I. Males and females were equally represented among individuals with the severe phenotype. There were more females than males (55 vs. 45%) among individuals with attenuated MPS I. The majority of individuals enrolled in the MPS I Registry were from either North America or Europe (50% and 43%, respectively). There was a 61.5% overlap among those with both length/height and head circumference. For the subpopulation of individuals with head circumference data (*n* = 450), the geographic distribution was similar to the total population; however, due to the lower age of those used in the head circumference analysis (0–36 months), the median age of diagnosis was lower in those with attenuated disease.

**Table 1:** Characteristics of untreated MPS I registrants with usable length/height measurements

	Severe MPS I		Attenuated MPS I	
	Male	Female	Male	Female
Registrants with usable^a^ initial measurements (*n*)	463		207	
	236	227	94	113
Number of usable records (*n*)	546	547	338	383
Natural history follow‐up time (years) (median [IQR])	1.4 (1.0,2.5)	1.4 (1.1,2.6)	9.1 (4.6,15.6)	9.4 (5.6,13.6)
Reported age at diagnosis (years) (median [IQR])	0.9 (0.6,1.3)	1.0 (0.7,1.5)	4.5 (3.2,6.6)	4.8 (2.3,8.3)
Registrant distribution				
North America (*n* [%])	119 (50.4)	125 (55.1)	50 (53.2)	46 (40.7)
Europe (*n* [%])	108 (45.8)	89 (39.2)	37 (39.4)	59 (52.2)
Latin America (*n* [%])	5 (2.1)	12 (5.3)	7 (7.4)	6 (5.3)
Asia‐Pacific (*n* [%])	<5	<5	<5	<5

*Note*: IQR = interquartile range (25%, 75%).

Abbreviations: IQR, interquartile range; MPS I, Mucopolysaccharidosis Type I.

a Defined as data with units and that were physiologically plausible.

### 3.2 Growth curves for severe MPS I

**Figure 1:**
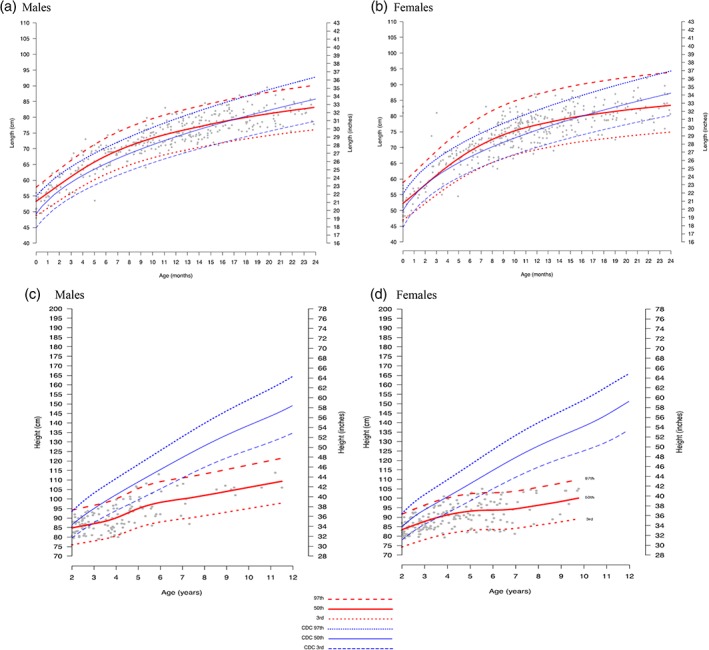
Estimated length/height curves for untreated individuals with severe MPS I disease, 0–24 months and 2–12 years old, for males (a, c) and females (b, d), with CDC/WHO standard curves overlaid on a scatter plot of MPS I Registry data. LMS estimated curves (red) and reference curves (blue) for length/height by age are shown overlaid on MPS I registry data (gray scatter) for individuals with severe MPS I). For those 0–24 months (a and b), (a): observations from 208 males with 410 records; (b): observations from 193 females and 367. For those 2–12 years (c and d): (c): observations from 58 males with 136 records; (d): observations from 68 females with 180 records. Median MPS I estimated length/height is shown as solid red lines, and the 97th and 3rd percentiles are shown as red hatched lines. CDC median and 97th and 3rd percentiles curves are shown in blue. Individual data points are shown as gray dots. CDC, Centers for Disease Control and Prevention; MPS I, Mucopolysaccharidosis Type I; LMS, Lambda Mu Sigma; WHO, World Health Organization

Figure 1a,b shows the estimated length versus age curves of untreated males (A) and females (B) from 0 to 24 months of age with severe MPS I. Growth patterns are similar between males and females and tend to follow the reference curves between 12 and 24 months. Increased length relative to the reference is observed for both untreated males and untreated females between 6 and 12 months of age. Growth curves through 12 years are shown for untreated males and females with severe disease in Figure 1c,d, respectively. By 4 years of age, estimated median height drops below the third percentile and remains below the reference curves. Figures S1 and S2 show WHO and CDC reference curves overlaid on scatter plots of natural history data for individuals with severe disease.

### 3.3 Growth curves for attenuated MPS I

**Figure 2:**
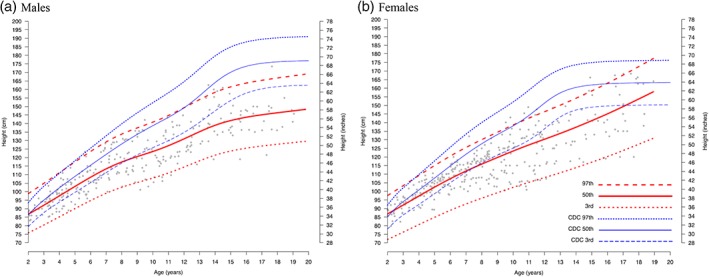
Estimated height curves for untreated individuals with attenuated MPS I disease 2–20 years old with CDC/WHO standard curves overlaid on a scatter plot of MPS I registry data. LMS estimated curves (red) and reference curves (blue) for height by age are shown overlaid on MPS I registry data (gray scatter) for those with attenuated MPS I. (a): observations from 87 males with 298 records; (b): observations from 104 females with 342 records. Median MPS I estimated length/height is shown as solid red lines, and the 97th and 3rd percentiles are shown as red hatched lines. CDC median and 97th and 3rd percentiles curves are shown in blue. CDC, Centers for Disease Control and Prevention; MPS I, Mucopolysaccharidosis Type I; LMS, Lambda Mu Sigma; WHO, World Health Organization

The median age of diagnosis of MPS I in the untreated attenuated cohort was 4.5 years in males and 4.8 years in females (Table 1). Figure 2a,b shows estimated growth curves and the corresponding age‐ and sex‐reference curves for untreated males and females with attenuated MPS I age from 2 to 20 years old. The MPS I registry‐generated height curves for the 97th, 50th (median), and 3rd centiles for untreated individuals with attenuated MPS I were overlaid on both the individual patient data points and the reference CDC curves showing 97th, 50th, and 3rd centiles. The estimated median height for untreated individuals with attenuated MPS I falls below the CDC third percentile by 9 years old, although divergence in height from the standard median growth curve is apparent beginning at 2 years of age. Growth in both untreated males and females with attenuated disease continues through age 18 but appears to slow in males at an earlier age than females (Figure 2a,b). There is a probable lack of pubertal growth spurts among untreated individuals with attenuated MPS I, especially in females, but additional data are needed for a complete determination. Scatter plots of natural history data for individuals with untreated attenuated disease overlaid on reference curves are shown in Figure S3. Few observations were available for the length of untreated MPS I patients with attenuated disease below 24 months of age (Figures S4 and S5).

### 3.4 Head circumference

**Figure 3:**
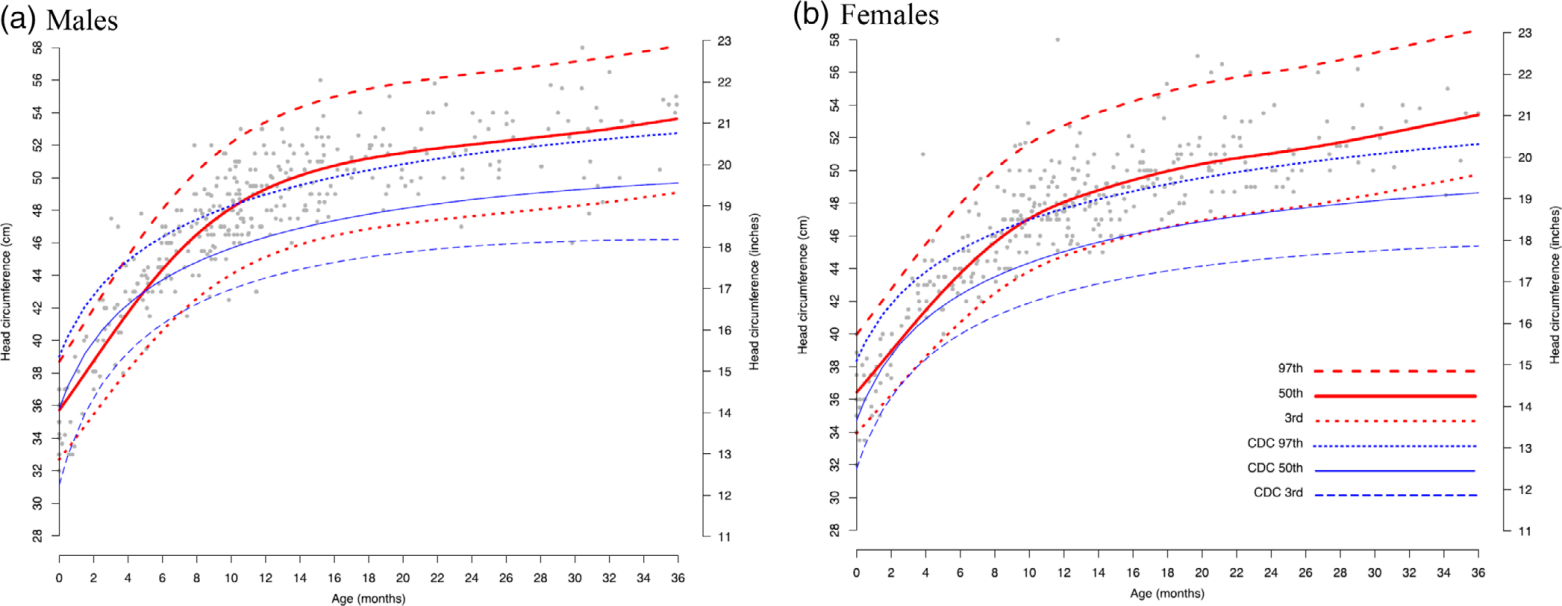
Head circumference estimates for untreated individuals with severe MPS I, 0–36 months old. These panels show, for males (a) and females (b) with severe MPS I, the LMS estimated head circumference (red) and reference curves (blue) by age overlaid on MPS I registry data (gray scatter). (a): 195 males with 366 records; (b): 191 females with 362 observations. Median MPS I estimated head circumference is shown as solid red lines, and the 97th and 3rd percentiles are shown as red hatched lines. CDC median and 97th and 3rd percentiles curves are shown in blue. CDC, Centers for Disease Control and Prevention; MPS I, Mucopolysaccharidosis Type I; LMS, Lambda Mu Sigma

Figure 3a,b shows the estimated head circumference of untreated males (A) and females (B) with MPS I from 0 to 36 months of age, overlaid on the CDC reference curves. Observations were plotted for 195 males with severe phenotype with 406 records, and 191 females with severe phenotype with a total of 401 records. Estimated median head circumferences larger than the CDC median curve are apparent by 5 months of age and remain above the standard curves for the duration of the observation period. The full set of CDC reference curves overlaid on scatter plots of MPS I data from the registry are shown in Figure S6.

There were few available head circumference data points for untreated individuals in the attenuated MPS I group (22 males and 24 females) in the 0–36 month interval, preventing meaningful interpretation of the data. Scatter plots and estimated curves are provided in Figures S7 and S8, respectively. The available data suggest that median head circumference in both sexes rises above the CDC median from 5 months through 3 years of age.

## 4 DISCUSSION

The MPS I registry data provide a unique source of natural history growth information on individuals who were not treated for the disease. Median body length for individuals with severe MPS I was slightly greater than the reference median in the first year. This is similar to observations in other MPS subtypes, including MPS II, MPS III, and MPS VI, where either normal growth or overgrowth is observed early in life in both severe and attenuated phenotypes (Montano et al., 2008; Muschol et al., 2019; Patel et al., 2014; Quartel et al., 2015; Rozdzynska‐Swiatkowska, Jurecka, Cieslik, & Tylki‐Szymanska, 2015; Tomatsu et al., 2012). Thus, short stature is not a common finding in the first 2 years of life for individuals with either severe or attenuated MPS I.

The median growth of individuals with untreated severe MPS I begins to deviate from the reference curves by approximately 6 months of age with a higher mean value and then falls below the third percentile of the reference curve by 4 years of age among both males and females. Limited studies in humans and extensive studies in MPS I animal models highlight the complexity of the underlying disease pathogenesis and indicate that the primary defect leads to both developmental impacts on the chondrocyte and growth plate as well as activation of complex pathogenic cascades. One possible explanation for disordered growth is that accumulation of GAGs in the epiphyseal plates directly disrupts the growth process. The study of a 30‐month old child with severe MPS I showed marked irregularities in chondrocyte orientation, disruption of the column architecture, and vacuolization with enlargement of the cellular border (Silveri, Kaplan, Fallon, Bayever, & August, 1991). In MPS I mice immunohistochemical analyses, increased GAGs in chondrocytes at the ossification zones are associated with altered FGF2 and Indian hedgehog distribution in growth plates (Kingma et al., 2016) and inhibition of collagenolytic activity of cathepsin K at the subepiphyseal growth plate (Wilson et al., 2009). In 5‐week growth plate cartilage in MPS I knock‐out mice in which no obvious morphologic changes are evident in bone, there were alterations in mRNA expression patterns of numerous genes indicating that instead of GAG accumulation near the growth plate there are complex alterations of extracellular matrix components early in disease progression that lead to disruption of chondrocyte maturation and proliferation at the growth plate (Heppner, Zaucke, & Clarke, 2015). These studies have provided insight into potential causes of short stature in MPS I; however, the pathophysiology underlying the patterns of growth has yet to be elucidated.

The median head circumference for untreated individuals with severe MPS I is well above the CDC reference curves by 3–4 months of age through 3 years of age. Previous studies have shown that individuals with untreated severe MPS I have head circumferences larger than population norms, but less is known about the attenuated phenotype (Aldenhoven et al., 2015; Kiely, Kohler, Coletti, Poe, & Escolar, 2017; Tylki‐Szymanska, Rozdzynska, Jurecka, Marucha, & Czartoryska, 2010). An analysis of 55 individuals with severe MPS I from a single center reported that enlarged head circumference was apparent at a median age of 8 months (Kiely et al., 2017). Thus, higher‐than‐normal OFC can serve as a clue to the diagnosis of MPS I, similar to males with MPS II (Parini, Jones, Harmatz, Giugliani, & Mendelsohn, 2016). Several cranial defects in MPS I likely contribute to altered head circumference, but it is not clear which contributes most. Even though thickened calvaria are noted in MPS I, macrocephaly is attributed to intracranial processes associated with megalencephaly, including accumulation of GAGs and sphingolipids in neurons, enlarged perivascular spaces, chronic communicating hydrocephalus, and neuroinflammation (Pavone et al., 2017). An initial report of a brain necropsy in MPS I identified swelling of most of the large cerebral cortical neurons with increased numbers of astrocytes, numerous perivascular aggregations of fibrous astrocytes throughout gray matter, and greatly enlarged perivascular spaces in the white matter filled with collagen and microglial phagocytes (Bishton, Norman, & Tingey, 1956). Brain assessments in MPS disorders also demonstrated a mesenchymal contribution to increased brain size that is due to mononuclear cells filled with GAGs in distended periadventitial spaces, GAG accumulation in leptomeninges, and, instead of GAGs, the neurons contained an excessive amount of glycolipid‐like material (Dekaban & Constantopoulos, 1977) shown to be an accumulation of sphingolipids (Constantopoulos & Dekaban, 1978; Kreutz et al., 2013). Brain MRIs of attenuated MPS I individuals demonstrate dilated perivascular spaces and enlargement of subarachnoid spaces in addition to increased size of supratentoral ventricles (Matheus et al., 2004).

There were several possible limitations of the study as it is a clinically based registry. Variation in length/height measurements due to interrater variability and complications inherent to MPS I (e.g., joint contracture) could contribute to errors. Some of this variation is compensated for by multiple observations from a single individual when possible. Due to the age at presentation of those with attenuated disease, usually over the age of 36 months, there were limited number of length and head circumference observations for this subset. The likelihood of the potential bias of joint contracture among those with attenuated disease in the natural history period is low as it tends to reflect the joints of the fingers and lower arms. This is because this symptom would likely merit the start of treatment. However, in the present analysis, the LMS method is used to account for this potential skewness by minimizing the sum of the squared errors (Cole, 1990). The authors acknowledge that this method is more robust with a larger sample size. Finally, the data collected from the registry represent primarily North America and Europe, potentially limiting generalizability to other regions, including Asia‐Pacific and Latin America. The registry is currently opening sites in other regions including Asia, Latin America, and the Middle East, which should enhance representation from other ethnic groups.

## 5 CONCLUSIONS

Even though there are limitations, growth curve assembly for individuals with MPS I may be helpful for the evaluation of long‐term outcomes of therapeutic interventions. While additional registry data continue to be collected in untreated individuals with MPS I, these growth curves could prove clinically beneficial in evaluating medical concerns unrelated to anticipated MPS I manifestations.

## AUTHOR CONTRIBUTIONS

D.V., L.C., L.B., H.K., J.M., and N.G. participated in the development and design of the study, interpretation of data, and drafting/revision of the manuscript. In addition, H.K. performed data analysis. All authors read and approved the final manuscript.

## DISCLOSURE OF INTERESTS

David Viskochil: Member of the International MPS I Registry advisory board, honoraria for speaking, consulting, and travel expenses from Sanofi Genzyme. Lorne A Clarke: Member of the International MPS I Registry advisory board, received speaker's fees for educational events related to lysosomal disease from Sanofi. Luisa Bay: Member of the International MPS I Registry advisory board, honoraria for speaking, consulting, and travel expenses from Sanofi Genzyme.

Nathalie Guffon: Member of the International MPS I Registry advisory board; participated in the MPS I Registry, serves on advisory boards for Sanofi Genzyme. Hillary Keenan: employee of Sanofi Genzyme. Joseph Muenzer: Serves on advisory boards and consult for Sanofi Genzyme and BioMarin. Member of the International MPS I Registry advisory board.

## Supporting information


**Figure S1** Scatter plots of length by age, 0–24 months old, of untreated individuals with severe MPS I from the MPS I Registry, on WHO/CDC standard curves. Panel A: 208 males with 410 records; panel B: 193 females with 367 records.
**Figure S2**. Scatter plots of height by age (years) of untreated individuals with severe MPS I from the MPS I Registry, 2–12 years old, on CDC standard curves. Panel A: 58 males with 136 records; panel B: 68 females with 180 records.
**Figure S3**. Scatter plots of height by age, 0–24 months old, of untreated individuals with attenuated MPS I from the MPS I Registry on WHO/CDC standard curves. Panel A: 20 males with 40 records; panel B: 19 females with 41 records.
**Figure S4**. Scatter plots of height by age, 2–20 years old, of untreated individuals with attenuated MPS I from the MPS I Registry on CDC standard curves. Panel A: 87 males with 298 records; panel B: 104 females with 342 records.
**Figure S5**. Estimated length for age for untreated individuals with attenuated MPS I disease, 0–24 months old, with CDC/WHO standard curves overlaid on a scatter plot of MPS I Registry data.
**Figure S6**. Scatter plots of head circumference by age, 0–36 months old, of untreated individuals with severe MPS I from the MPS I Registry on CDC standard curves. Panel A: 195 males with 366 records; panel B: 191 females with 362 records.
**Figure S8**. Head circumference estimates for untreated individuals with attenuated MPS I, 0–36 months old.
These panels show, for males (A) and females (B) with severe MPS I, the LMS estimated head circumference (red) and reference curves (blue) by age overlaid on MPS I Registry data (gray scatter). Panel A: 22 males with 40 records; panel B: 24 females with 39 observations. Median MPS I estimated head circumference is shown as solid red lines, and the 97th and third percentiles are shown as red hatched lines. CDC median and 97th and third percentiles curves are shown in blue.

## Data Availability

The data that support the findings of this study can be requested by MPS I Registry participants through a MPS I Registry Data Analyses Request form. The data are not publicly available due to privacy or ethical restrictions. For additional information, please contact rarediseaseregistries@sanofi.com.
