# Supplementary material for: Growth patterns for untreated individuals with MPS I: Report from the international MPS I registry
Source: Am J Med Genet A. 2019 Oct 22;179(12):2425–32. doi: 10.1002/ajmg.a.61378 (PMC6899772; doi:10.1002/ajmg.a.61378)

**Supplementary Figures**

**Figure S1**. Scatter plots of length by age, 0-24 months old, of untreated individuals with severe MPS I from the MPS I Registry, on WHO/CDC standard curves. Panel A: 208 males with 410 records; panel B: 193 females with 367 records.

A) Males B) Females

**Figure S2.** Scatter plots of height by age (years) of untreated individuals with severe MPS I from the MPS I Registry, 2-12 years old, on CDC standard curves. Panel A: 58 males with 136 records; panel B: 68 females with 180 records.

A) Males B) Females

**Figure S3.** Scatter plots of height by age, 0-24 months old, of untreated individuals with attenuated MPS I from the MPS I Registry on WHO/CDC standard curves. Panel A: 20 males with 40 records; panel B: 19 females with 41 records.

A) Males B) Females


**Figure S4.** Scatter plots of height by age, 2-20 years old, of untreated individuals with attenuated MPS I from the MPS I Registry on CDC standard curves. Panel A: 87 males with 298 records; panel B: 104 females with 342 records.

A) Males B) Females

**Figure S5.** Estimated length for age for untreated individuals with attenuated MPS I disease, 0-24 months old, with CDC/WHO standard curves overlaid on a scatter plot of MPS I Registry data.

LMS estimated curves (red) and reference curves (blue) for height by age are shown overlaid on MPS I Registry data (gray scatter) for those with attenuated MPS I. Panel A: observations from 20 males with 40 records; panel B: observations from 19 females with 41 records. Median MPS I estimated length is shown as solid red lines, and the 97th and 3rd percentiles are shown as red hatched lines. CDC median and 97th and 3rd percentiles curves are shown in blue.

A) Males B) Females


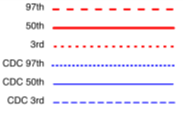
**Figure S6.** Scatter plots of head circumference by age, 0-36 months old, of untreated individuals with severe MPS I from the MPS I Registry on CDC standard curves. Panel A: 195 males with 366 records; panel B: 191 females with 362 records.

A) Males B) Females

**Figure S7.** Scatter plots of head circumference by age, 0-36 months old, of untreated individuals with attenuated MPS I from the MPS I Registry on CDC standard curves. Panel A: 22 males with 40 records; panel B: 24 females with 39 records.

A) Males B) Females

**Figure S8.** Head circumference estimates for untreated individuals with attenuated MPS I, 0-36 months old.

These panels show, for males (A) and females (B) with severe MPS I, the LMS estimated head circumference (red) and reference curves (blue) by age overlaid on MPS I Registry data (gray scatter). Panel A: 22 males with 40 records; panel B: 24 females with 39 observations. Median MPS I estimated head circumference is shown as solid red lines, and the 97th and 3rd percentiles are shown as red hatched lines. CDC median and 97th and 3rd percentiles curves are shown in blue.

A) Males B) Females

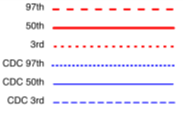

Supplement: Supplementary file 1 — Figure S1 Scatter plots of length by age, 0–24 months old, of untreated individuals with severe MPS I from the MPS I Registry, on WHO/CDC standard curves. Panel A: 208 males with 410 records; panel B: 193 females with 367 records. Figure S2. Scatter plots of height by age (years) of untreated individuals with severe MPS I from the MPS I Registry, 2–12 years old, on CDC standard curves. Panel A: 58 males with 136 records; panel B: 68 females with 180 records. Figure S3. Scatter plots of height by age, 0–24 months old, of untreated individuals with attenuated MPS I from the MPS I Registry on WHO/CDC standard curves. Panel A: 20 males with 40 records; panel B: 19 females with 41 records. Figure S4. Scatter plots of height by age, 2–20 years old, of untreated individuals with attenuated MPS I from the MPS I Registry on CDC standard curves. Panel A: 87 males with 298 records; panel B: 104 females with 342 records. Figure S5. Estimated length for age for untreated individuals with attenuated MPS I disease, 0–24 months old, with CDC/WHO standard curves overlaid on a scatter plot of MPS I Registry data. Figure S6. Scatter plots of head circumference by age, 0–36 months old, of untreated individuals with severe MPS I from the MPS I Registry on CDC standard curves. Panel A: 195 males with 366 records; panel B: 191 females with 362 records. Figure S8. Head circumference estimates for untreated individuals with attenuated MPS I, 0–36 months old. These panels show, for males (A) and females (B) with severe MPS I, the LMS estimated head circumference (red) and reference curves (blue) by age overlaid on MPS I Registry data (gray scatter). Panel A: 22 males with 40 records; panel B: 24 females with 39 observations. Median MPS I estimated head circumference is shown as solid red lines, and the 97th and third percentiles are shown as red hatched lines. CDC median and 97th and third percentiles curves are shown in blue. [file AJMG-179-2425-s001.docx]
